# Histone demethylase KDM6B inhibits breast cancer metastasis by regulating Wnt/β‐catenin signaling

**DOI:** 10.1002/2211-5463.13236

**Published:** 2021-07-12

**Authors:** Jing Xun, Ruifang Gao, Botao Wang, Yifan Li, Yuan Ma, Jun Guan, Qi Zhang

**Affiliations:** ^1^ Tianjin Key Laboratory of Acute Abdomen Disease Associated Organ Injury and ITCWM Repair Institute of Acute Abdominal Diseases Tianjin Nankai Hospital China; ^2^ Tianjin Institute of Medical & Pharmaceutical Sciences Tianjin China; ^3^ Graduate School of Tianjin Medical University China; ^4^ Tianjin Key Laboratory of Acute Abdomen Disease Associated Organ Injury and ITCWM Repair Institute of Acute Abdominal Diseases, Integrated Chinese and Western Medicine Hospital Tianjin University China

**Keywords:** breast cancer, KDM6B, metastasis, Wnt/β‐catenin signaling

## Abstract

Tumor metastasis remains a major challenge for patients with breast cancer. Aberrant epigenetic factor lysine‐specific demethylase 6B (KDM6B) has been associated with tumor progression. Here, we show that KDM6B is significantly down‐regulated in human breast cancer tissues, and its low expression is associated with poor prognosis of patients with breast cancer. Furthermore, overexpression of KDM6B remarkably inhibited cell proliferation, invasion, migration and epithelial–mesenchymal transition markers of breast cancer cells *in vitro* and tumor growth and lung metastasis *in vivo*. Notably, the expression of KDM6B in breast cancer tissues was negatively correlated with that of β‐catenin, and overexpression of KDM6B decreased the expression of β‐catenin and its accumulation in the nucleus of breast cancer cells. Overall, our findings provide novel insights into suppression of metastasis of breast cancer cells by KDM6B via β‐catenin and suggest involvement of the KDM6B‐Wnt/β‐catenin axis in breast cancer progression.

## Graphical abstract

We investigated the effect of histone demethylase lysine‐specific demethylase 6B (KDM6B) on metastasis of breast cancer. KDM6B could inhibit the growth and metastasis of breast cancer by suppressing β‐catenin expression and its nuclear accumulation. These results suggest a significant role for the KDM6B‐Wnt/β‐catenin axis in breast cancer progression.

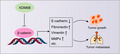

**Abbreviations**
CCK‐8: Cell Counting Kit‐8
EMT: epithelial–mesenchymal transition
H3K27: histone H3 lysine 27
KDM6B: lysine‐specific demethylase 6B
MMP: matrix metalloproteinase
TCGA: The Cancer Genome Atlas

Breast cancer is the most common malignancy and the leading cause of cancer‐related death among women all over the world [1]. Despite the therapeutic management, including surgical section, chemoradiotherapy and biological therapy, having been improved over the past several decades, metastasis remains incurable and is the main cause of death in patients with breast cancer. Therefore, novel molecules that regulate breast cancer metastasis should be identified to promote the development of antimetastasis strategies.

Histone methylation, one of the epigenetic modifications, occurs in lysine and arginine residues that have been linked to transcriptional regulation, cell fate determination, terminal differentiation, and X inactivation. Moreover, aberrant histone modification has been shown to integrate with transcription factors to regulate tumor progression. The methylation of histone H3 lysine 27 (H3K27) is an important epigenetic modification and is associated with gene repression that is mediated by the polycomb repressive complex 2. Lysine‐specific demethylase 6B (KDM6B), also known as Jumonji domain‐containing protein 3, is a member of the histone demethylases. It specifically catalyzes the removal of the trimethyl group from H3K27 lysine residue to active chromatin conformation [2, 3]. It has been shown that KDM6B plays essential roles in development, differentiation, cell senescence and inflammation [4, 5, 6]. Moreover, the dysregulation of KDM6B expression or activity is involved in carcinogenesis. For example, KDM6B acts as a critical driver of hepatocellular carcinoma stem cell‐like and metastatic behaviors. In addition, KDM6B has been shown to be overexpressed in gastric cancer, hepatocellular carcinoma and lymphoma and promote their survival and progression [7, 8, 9]. However, KDM6B is a proposed tumor suppressor in oncogene‐induced senescence, colorectal cancer and pancreatic ductal adenocarcinoma [10, 11, 12]. Our previous study showed that KDM6B inhibited the stem cell‐like properties of breast cancer by inhibiting the expression of stemness‐related transcription factor OCT4 [13]. All these findings suggest an important role of KDM6B in the development of cancers.

The Wnt/β‐catenin pathway is one of the most common dysfunctional signaling pathways in human malignancies. This pathway participates in the initiation, epithelial–mesenchymal transition (EMT) and progression of different cancers [14]. It has been reported that KDM6B is associated with Wnt/β‐catenin signaling. KDM6B controls mesodermal and cardiovascular differentiation of embryonic stem cells via facilitating the recruitment of β‐catenin [15]. KDM6B regulates the expression of Wnt target gene c‐Myc in hematopoietic stem cells [16]. Vitamin D3 analogue 1,25(OH)2D3 induces KDM6B expression and interferes with Wnt/β‐catenin signaling in human colon carcinoma cells [17], which implies a relationship between KDM6B and Wnt/β‐catenin signaling.

In this study, we verified that KDM6B was down‐regulated in human breast cancer tissues, and low KDM6B expression was associated with poor prognosis of patients with breast cancer. Furthermore, overexpression of KDM6B remarkably inhibited cell proliferation, invasion, migration and EMT markers of breast cancer cells *in vitro* and lung metastasis *in vivo*. In addition, we found that the expression of KDM6B in breast cancer tissues was negatively correlated with that of β‐catenin, and overexpression of KDM6B decreased the expression of β‐catenin and its accumulation in the nucleus of breast cancer cells. Our findings suggest that KDM6B inhibits the metastasis of breast cancer probably by regulating Wnt/β‐catenin signaling, which provides a novel mechanistic role of KDM6B in breast cancer metastasis.

## Materials and methods

### Cell culture

The human breast cancer cell lines MCF7, T47D, SKBR3, MDA‐MB‐468 and MDA‐MB‐231 were cultured in high‐glucose Dulbecco's modified Eagle's medium (Biological Industries, Kibbutz Beit HaEmek, Israel) containing 10% FBS (Biological Industries). Cells were maintained at 37 °C in a humidified atmosphere with 5% CO_2_.

### Vector construction and stable cell line establishment

For gene overexpression, the DNA sequence encoding human KDM6B was PCR amplified from the pCMV‐HA‐KDM6B plasmid (Addgene, Cambridge, MA, USA) and cloned into the pLV‐EF1α‐MCS (multiple cloning site)‐IRES‐Bsd plasmid (Biosettia, San Diego, CA, USA). To establish a stable KDM6B expression cell line, we infected MDA‐MB‐231 cells with pLV‐EF1α‐KDM6B‐IRES‐Bsd using a lentivirus transfection system according to the manufacturer's instructions (Biosettia).

### RNA isolation and real‐time quantitative PCR

Total RNA was isolated from breast cancer cells using TRIzol reagent (Invitrogen, Carlsbad, CA, USA). Reverse transcription was performed using the TransScript First‐Strand cDNA Synthesis SuperMix Kit (TransGen Biotech, Beijing, China) according to the manufacturer's recommendations. The primers used were as follows: homo‐KDM6B, forward: 5′‐agcaaacgggatgccttctca‐3′ and reverse: 5′‐tgttcgccactcgcttccaccag‐3′; homo‐matrix metalloproteinase‐2 (homo‐MMP‐2), forward: 5′‐tgatggcatcgctcagatcc‐3′ and reverse: 5′‐ggcctcgtataccgcatcaa‐3′; homo‐MMP‐7, forward: 5′‐catgattggctttgcgcgag‐3′ and reverse: 5′‐agactgctaccatccgtcca‐3′; homo‐MMP‐9, forward: 5′‐tctatggtcctcgccctgaa‐3′ and reverse: 5′‐catcgtccaccggactcaaa‐3′; homo‐β‐actin, forward: 5′‐cagaaggagattactgctctggct‐3′ and reverse: 5′‐tactcctgcttgctgatccacatc‐3′.

### Western blotting

Cell lysates were prepared with radioimmunoprecipitation assay buffer, and the protein concentrations of the lysates were determined by bicinchoninic acid assay (Thermo Fisher Scientific, Waltham, MA, USA). Equivalent samples were loaded onto 10% SDS/PAGE gels and transferred to nitrocellulose membranes. After blocking nonspecific binding sites, the membranes were incubated overnight at 4 °C with the appropriate antibodies, including anti‐KDM6B (ab38113; Abcam, Cambridge, MA, USA), β‐catenin (93790; Cell Signaling Technology, Danvers, MA, USA) and β‐actin (sc‐16632; Santa Cruz Biotechnology, Santa Cruz, CA, USA). After incubation with horseradish peroxidase‐conjugated secondary antibodies, proteins were detected using an enhanced chemiluminescence kit (Millipore, Billerica, MA, USA).

### Proliferation assay

Cell proliferation was determined using the Transdetect Cell Counting Kit (Transgene, Beijing, China). Cells were seeded in triplicate in 96‐well plates. After cell adherence, 10 μL of Cell Counting Kit‐8 (CCK‐8) solution was added to each well, and then the cells were incubated for 2 h. Cell proliferation was measured at a wavelength of 450 nm using a microplate reader.

### Transwell migration assay

A total of 1 × 10^5^ cells were suspended in serum‐free medium and seeded into the upper Transwell chamber (8‐μm pore size, 24‐well plate). The bottom chamber contained 500 μL of medium containing 10% FBS. After incubation for 48 h, the inserts were fixed with 4% paraformaldehyde and stained with 0.5% crystal violet staining solution. Nonmigrated cells were removed from the upper chamber with cotton swabs, and the number of cells that migrated and attached to the bottom dishes was recorded using an OLYMPUS microscope (Olympus, Tokyo, Japan). Migrated cells from five random image fields were counted.

### Wound healing assay

A total of 1 × 10^6^ cells were seeded into a six‐well plate. A ‘wound’ was generated by a 10‐μL pipette tip when cells became confluent, and the cells were cultured with low serum (2% FBS). The wound healing process was recorded at 0, 24 and 48 h after wound using an OLYMPUS microscope. Migration distance was determined using imagej software [National Institutes of Health (NIH), Bethesda, MD, USA] as an average of the closed area relative to the initial wound area.

### Immunohistochemistry

The detailed procedures of immunohistochemical staining were described previously [18]. In brief, paraffin‐embedded sections were incubated with primary antibody and biotinylated secondary antibody, followed by an avidin–peroxidase complex. The sections were visualized with diaminobenzidine and counterstained with hematoxylin. Five random fields were obtained with a BX53 research microscope (Olympus).

### Immunofluorescence staining

Cells were grown on glass coverslips and fixed with cold methanol as previously described [19]. An antibody against E‐cadherin (610182; BD Transduction Laboratories, San Jose, CA, USA), β‐catenin (93790; CST), was used overnight at 4 °C and subsequently incubated with Alexa Fluor 488‐labeled secondary antibody (Invitrogen) at room temperature for 1 h. 4′,6‐Diamidino‐2‐phenylindole was used for counterstaining of the nucleus. Photographs were taken with a TCS‐SP5 fluorescence confocal microscope (Leica Microsystems, Mannheim, Germany). The fluorescence intensity was analyzed by imagej software.

### Animal models

KDM6B‐overexpressing or control MDA‐MB‐231 cells (1 × 10^6^) were injected into the mammary fat pad of 6‐ to 8‐week‐old female nude mice (*n* = 5 per group). Tumor volume was measured twice a week. The mice were sacrificed 2 months after injection. Tumor weight was measured, and the lung was embedded in paraffin and sliced for hematoxylin and eosin staining to detect metastasis. All animal experiments were performed strictly under the guidelines for laboratory animals of Tianjin Nankai Hospital, and the study was approved by the ethics committee of Tianjin Nankai Hospital.

### Statistical analysis

All data were analyzed using graphpad prism 8 software (GraphPad Software, San Diego, CA, USA). The Kaplan–Meier method was used for survival analysis. The results are shown as the mean ± SEM, and statistical significance was determined by two‐tailed Student's *t*‐test or two‐way ANOVA. The results were considered statistically significant at *P < 0.05, **P < 0.01, and ***P < 0.001.

## Results

### KDM6B is expressed at low levels and is correlated with patient survival in breast cancer

**Fig. 1:**
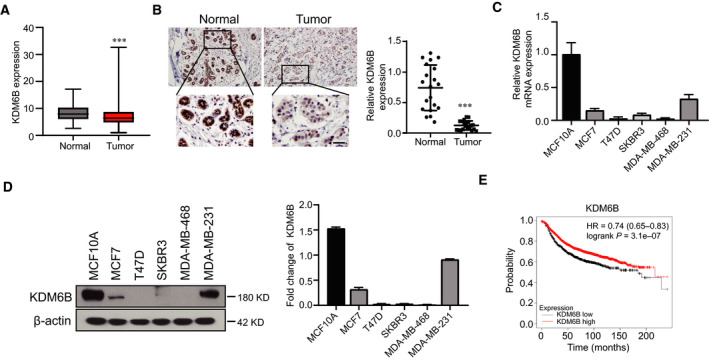
KDM6B is expressed at low levels and correlated with patient survival in breast cancer. (A) Analysis of KDM6B expression in breast cancer and normal tissues by TCGA database. (B) Immunohistochemical staining and the statistical result of KDM6B protein expression in 20 pairs of normal and breast cancer tissues (scale bar: 100 μm). (C) The mRNA levels of KDM6B were analyzed in different breast cancer cells by real‐time quantitative PCR. MCF‐10A cells served as a control. (D) The protein levels of KDM6B were analyzed by western blot in different breast cancer cells versus MCF‐10A cells. The right panel was the statistical result. (E) Kaplan–Meier overall survival curve of patients with breast cancer based on KDM6B expression (*n* = 3951). Data are shown as the mean ± SEM of three independent experiments. Student's *t*‐test was performed by using graphpad prism 8.0. ***P < 0.001.

To understand the expression of KDM6B in breast cancer, we explored The Cancer Genome Atlas (TCGA; https://www.cancer.gov/about‐nci/organization/ccg/research/structural‐genomics/tcga), a free database that includes the genomic, epigenomic, transcriptomic and proteomic data of 33 types of cancers and matched normal samples, to determine whether abnormal KDM6B expression was present. The results showed that KDM6B expression was significantly reduced in breast cancer compared with normal subjects (Fig. 1A). To confirm the earlier‐mentioned data analysis, we immunohistochemically stained biopsy samples from patients with breast cancer, which include 20 pairs of normal and breast cancer tissues. The results confirmed that the expression of KDM6B decreased in human breast cancer tissues (Fig. 1B). Consistently, real‐time quantitative PCR and western blotting assays in five breast cancer cell lines indicated that a sharp decrease in KDM6B mRNA and protein levels occurred compared with mammary MCF10A cells (Fig. 1C,D). More importantly, Kaplan–Meier analysis suggested that the overall survival rate of patients with breast cancer with low KDM6B expression was poorer than that of patients with high KDM6B expression (Fig. 1E). Taken together, these results indicate that KDM6B may play a suppressing role in breast cancer.

### KDM6B overexpression inhibits the proliferation capacity of breast cancer cells

**Fig. 2:**
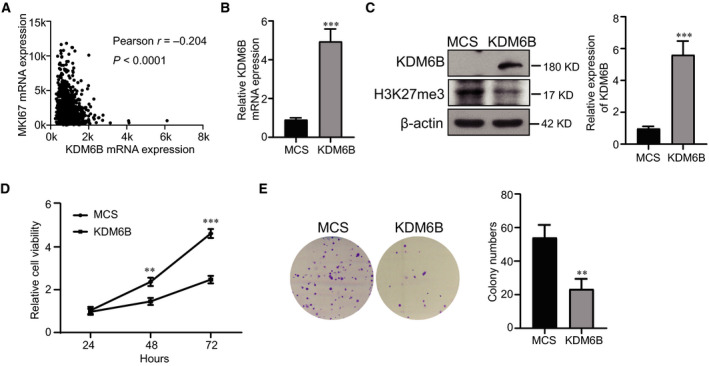
KDM6B overexpression inhibits the proliferation of breast cancer cells *in vitro*. (A) Correlation analysis between KDM6B and KI67 expression in breast cancer using TCGA dataset. (B) Real‐time quantitative PCR analysis of KDM6B overexpression efficacy in MDA‐MB‐231 cells. (C) Western blot analysis of KDM6B overexpression efficiency and H3K27me3 expression in MDA‐MB‐231 cells. (D) CCK‐8 assay analysis of the proliferation of KDM6B‐overexpressing MDA‐MB‐231 cells and control cells. (E) Colony formation assay analysis of the growth of breast cancer cells. Data shown represent the mean values ± SEM of three independent experiments. Student's *t*‐test was performed by using graphpad prism 8.0. **P < 0.01; ***P < 0.001.

To test the carcinogenic activity of KDM6B in breast cancer, we analyzed the relationship between the expression of KDM6B and KI67 using TCGA dataset. The results showed that the expression of KDM6B was negatively correlated with that of KI67 in breast cancer (Fig. 2A). Next, we stably overexpressed KDM6B in MDA‐MB‐231 cells via a lentivirus system. The level of KDM6B was verified by real‐time quantitative PCR and western blot analysis (Fig. 2B,C). Correspondingly, the expression of H3K27me3 at protein level was decreased (Fig. 2C). CCK‐8 and colony formation assays were performed to explore the effect of KDM6B on the proliferation of breast cancer cells. The results showed that the viability and number of colonies in KDM6B‐overexpressing MDA‐MB‐231 cells decreased significantly compared with those in control cells (Fig. 2D,E). These results indicate that KDM6B ectopic expression decreases the proliferation capacity of breast cancer cells *in vitro*.

### KDM6B overexpression impedes the migration capacity of breast cancer cells *in vitro*

**Fig. 3:**
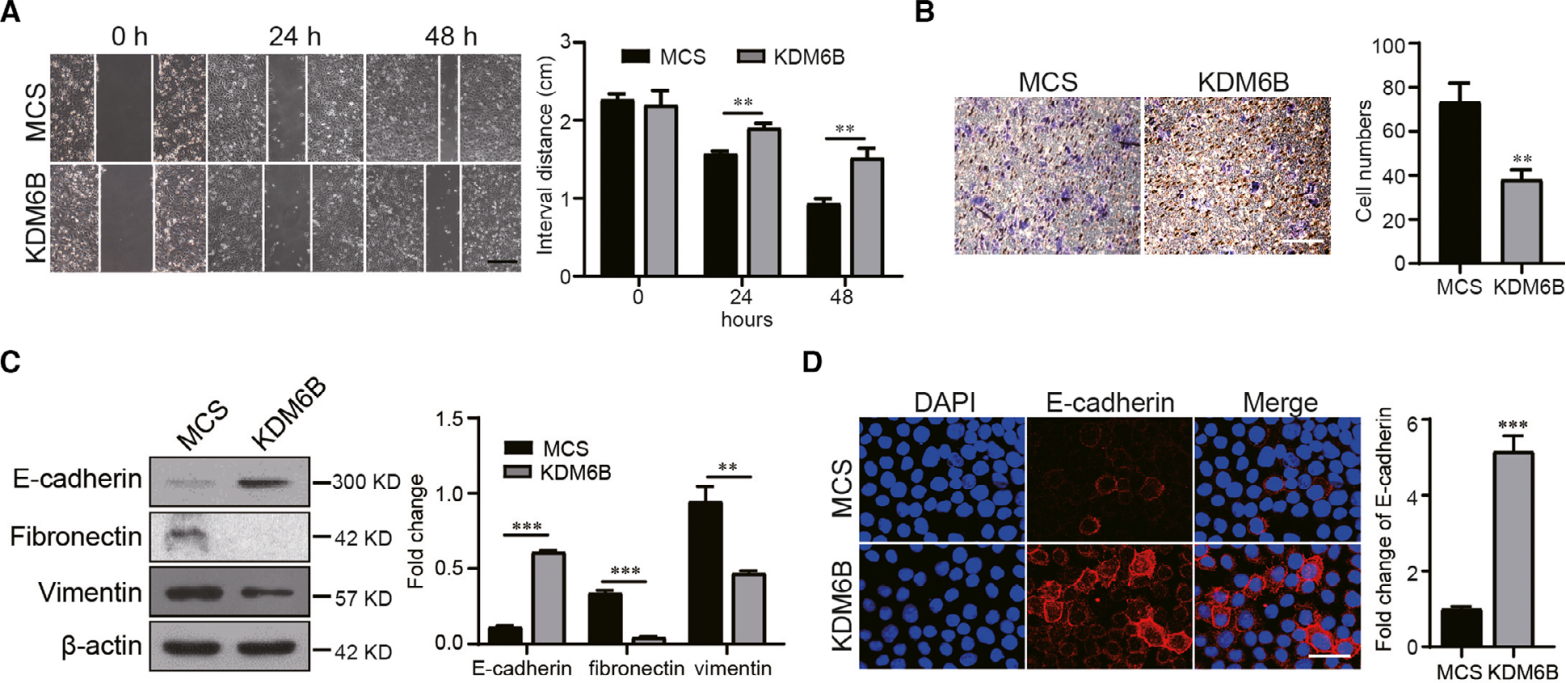
KDM6B overexpression impedes the migration capacity of breast cancer cells *in vitro*. (A) Representative images of the wound healing assay and statistical results of the migration distance are shown (scale bar: 100 μm). (B) Transwell assay of migrated cells and statistical results in KDM6B‐overexpressing MDA‐MB‐231 cells compared with vector controls (MCS; scale bar: 100 μm). (C) The analysis and statistical results of E‐cadherin, Fibronectin and Vimentin expression in KDM6B‐overexpressing MDA‐MB‐231 cells by western blot. (D) Immunofluorescence images and statistical results of E‐cadherin in KDM6B‐overexpressing T47D cells and control cells (scale bar: 200 μm). Data shown represent the mean values ± SEM of three independent experiments. Student's *t*‐test was performed by using graphpad prism 8.0. **P < 0.01; ***P < 0.001.

To investigate whether KDM6B is involved in the regulation of migration and invasion of breast cancer cells, we initially performed wound healing assays. The results showed that gap filling was significantly retarded in KDM6B‐overexpressing MDA‐MB‐231 cells compared with control cells (Fig. 3A). The same results were also confirmed by Boyden chamber experiment assays, which represent another method to test the migration and metastasis capacity of cells. We found that the number of migrated cells decreased in KDM6B‐overexpressing MDA‐MB‐231 cells versus vector control (Fig. 3B). These results suggested that KDM6B overexpression may suppress the metastasis of breast cancer cells. Because EMT is an important process during tumor metastasis, we subsequently tested the regulatory effect of KDM6B on EMT markers. Western blotting showed that the protein expression level of the key epithelial marker E‐cadherin increased and the mesenchymal markers fibronectin and vimentin decreased in KDM6B‐overexpressing MDA‐MB‐231 cells compared with control cells (Fig. 3C). The expression of E‐cadherin was further supported by immunofluorescence staining results in KDM6B‐overexpressing T47D cells (Fig. 3D). Our findings collectively support that KDM6B overexpression inhibits the migration and metastasis of breast cancer cells *in vitro*.

### KDM6B overexpression inhibits the growth and lung metastasis of breast cancer cells *in vivo*

**Fig. 4:**
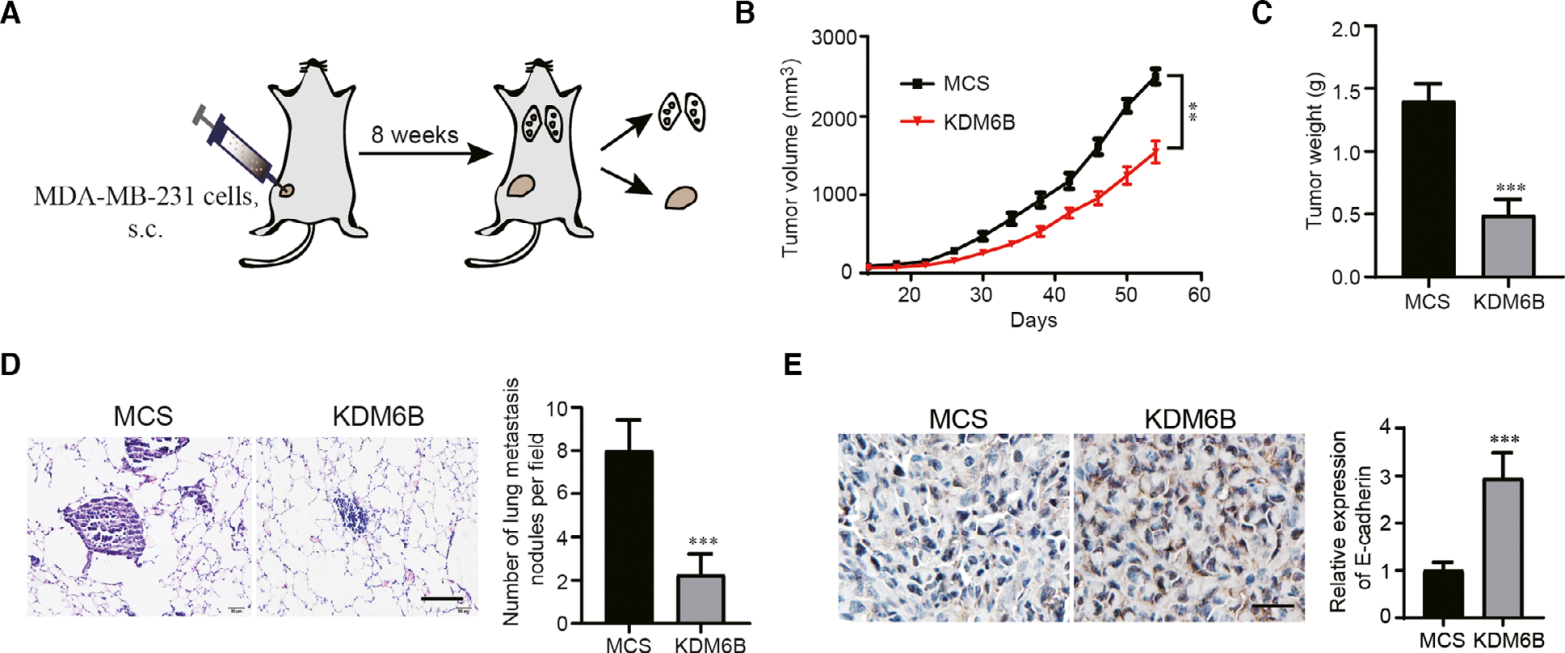
KDM6B overexpression inhibits tumor growth and metastasis of breast cancer *in vivo*. A mouse model was established for breast cancer via orthotopic implantation of MDA‐MB‐231 cells overexpressing KDM6B or MCS. (A) Schematic diagram of the mouse model. (B, C) Statistical results of tumor volume (B) and tumor weight (C). (D) Representative images and statistical results of lung metastasis (scale bar: 50 μm). (E) Representative images of E‐cadherin immunohistochemical staining in tumor tissues of a breast cancer mouse model (scale bar: 100 μm). Data are shown as the mean ± SEM of three independent experiments. Student's *t*‐test was performed by using graphpad prism 8.0. **P < 0.01; ***P < 0.001. s.c., subcutaneous.

To evaluate the impact of KDM6B on tumor growth and dissemination *in vivo*, we used an orthotopic xenograft mouse model. KDM6B‐overexpressing MDA‐MB‐231 cells and control cells were injected into the fourth fat pad of 6‐ to 8‐week‐old nude female mice (Fig. 4A). The mice were sacrificed, and tumor and lung tissues were obtained for identification of tumor growth and metastatic nodules in the lung. We found that KDM6B‐overexpressing MDA‐MB‐231 breast cancer cells grew significantly smaller in terms of volume and weight than the controls (Fig. 4B,C). Moreover, there were significantly fewer metastatic lung nodules in mice injected with KDM6B‐overexpressing MDA‐MB‐231 cells than in the controls (Fig. 4D). In addition, we also detected the expression of E‐cadherin in tumor tissue of an orthotopic xenograft mouse model by immunochemical staining. The results showed that compared with the MCS group, the expression of E‐cadherin decreased in the group of KDM6B overexpression (Fig. 4E). These results collectively suggest that KDM6B plays a suppressive role in the growth and metastasis of breast cancer *in vivo*.

### KDM6B overexpression suppresses the expression of β‐catenin in breast cancer cells

**Fig. 5:**
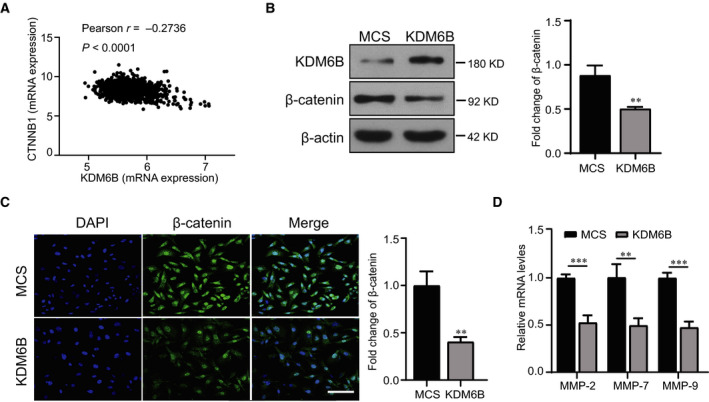
KDM6B overexpression suppresses the expression of β‐catenin in breast cancer cells. (A) Correlation analysis between KDM6B and β‐catenin expression in breast cancer using TCGA dataset. (B) The analysis and statistical results of β‐catenin expression in KDM6B‐overexpressing MDA‐MB‐231 cells by western blot. (C) Immunofluorescence images and statistical results of β‐catenin in KDM6B‐overexpressing T47D cells and control cells (scale bar: 200 μm). (D) Real‐time quantitative PCR analysis of MMP‐2, MMP‐7 and MMP‐9 expression in KDM6B‐overexpressing MDA‐MB‐231 cells. Data are shown as the mean ± SEM of three independent experiments. Student's *t*‐test was performed by using graphpad prism 8.0. **P < 0.01; ***P < 0.001. We investigated the effect of histone demethylase KDM6B on metastasis of breast cancer. KDM6B could inhibit the growth and metastasis of breast cancer by suppressing β‐catenin expression and its nuclear accumulation. These results suggest a significant role for the KDM6B‐Wnt/β‐catenin axis in breast cancer progression.

It has been known that the Wnt/β‐catenin signaling pathway plays a critical role in cell proliferation, differentiation and migration. Therefore, we next examined whether KDM6B regulates the metastasis of breast cancer via Wnt/β‐catenin signaling. We first analyzed the relationship between the expression of KDM6B and β‐catenin using TCGA dataset. The results showed that the expression of KDM6B was negatively correlated with that of β‐catenin in breast cancer (Fig. 5A). This correlation was then confirmed by KDM6B overexpression in MDA‐MB‐231 cells, in which the expression of β‐catenin was down‐regulated (Fig. 5B). Moreover, the accumulation of β‐catenin in the nucleus decreased in KDM6B‐overexpressing MDA‐MB‐231 cells (Fig. 5C). In addition, we also detected the target genes of the Wnt/β‐catenin signaling pathway. The results showed that overexpression of KDM6B decreased the mRNA levels of MMP‐2, MMP‐7 and MMP‐9 in MDA‐MB‐231 cells (Fig. 5D). All these data suggested that overexpression of KDM6B inhibited proliferation, migration and metastasis of breast cancer cells probably regulating the Wnt/β‐catenin signaling pathway.

## Discussion

Histone modifications play important roles in cell fate determination, terminal differentiation and X inactivation. KDM6B, a member of Jumonji domain‐containing proteins, acts as a histone demethylase and is an important regulator in multiple cellular processes, such as cell development, differentiation, senescence and inflammation. More importantly, a large body of data has shown the association of KDM6B abnormality with tumor progression [2]. Our previous study also showed that KDM6B inhibited the stem cell‐like properties of breast cancer by inhibiting the expression of OCT4. All these findings suggest an important role of KDM6B in cancer progression.

In this study, our data provided evidence that KDM6B expression decreased significantly in breast cancer tissues compared with normal tissues, which confirmed our TCGA dataset analysis. Moreover, we found the inhibitory role of KDM6B in cell proliferation, invasion and migration *in vitro*, suggesting a suppressive role of KDM6B in tumor growth and metastasis of breast cancer. This was further verified by an orthotopic xenograft mouse model of breast cancer.

Metastasis is a multistep process, and EMT has been shown to promote tumor metastasis. A previous study reported that aberrant KDM6B expression up‐regulates Slug to induce EMT, invasive migration, stem cell‐like traits and metastatic properties in hepatocellular carcinoma [8], although KDM6B plays a permissive role in TGF‐β‐induced EMT in mammary epithelial cells by stimulating Snail expression [20]. In our study, high expression of KDM6B inhibited EMT of breast cancer cells, which was confirmed in the xenografts. In support of our findings, Pereira *et al*. [12] reported that Jumonji domain‐containing protein 3 negatively regulates Snail and EMT in colon cancer by mediating vitamin D signaling. This suggested that KDM6B regulated the EMT process depending on the cancer type or the stimuli of the microenvironment.

More importantly, a previous study has shown that the Wnt/β‐catenin signaling pathway regulates EMT of cancer cells [21]. In addition, MMP‐2, MMP‐7 and MMP‐9, the target genes of Wnt/β‐catenin signaling, function to degrade extracellular matrix and basement membrane to promote tumor cell metastasis [22]. Xu *et al*. [23] found that breast cancer tissues have significantly lower expression of KDM6B, and the positivity rates of KDM6B, MMP‐2 and VEGF in breast cancer tissues were significantly correlated with tumor progression. However, the regulatory mechanism remains unclear. In this study, we found that KDM6B expression was negatively correlated with β‐catenin expression in breast cancer using TCGA dataset analysis. Furthermore, overexpression of KDM6B decreased the expression of β‐catenin and its accumulation in the nucleus of breast cancer cells. It was also confirmed that overexpression of KDM6B decreased the target genes of the Wnt/β‐catenin signaling pathway. These results suggested that the KDM6B/β‐catenin axis might be one of the molecular mechanisms inhibiting the Wnt signaling pathway in breast cancer. However, how KDM6B regulates Wnt/β‐catenin signaling needs to be further explored. Some of our data tend to suggest that KDM6B may depend on the ubiquitination to regulate β‐catenin expression. The molecular regulatory mechanism will be studied further.

Taken together, our results provided the support that KDM6B was lowly expressed in breast cancer, and the low‐level expression of KDM6B was closely associated with poor prognosis of patients with breast cancer. In addition, KDM6B overexpression repressed proliferation, invasion, migration of breast cancer cells *in vitro* and metastasis *in vivo*. Moreover, KDM6B down‐regulated the expression of β‐catenin and its accumulation in the nucleus of breast cancer cells. Our findings implied a novel molecular mechanism that overexpression of KDM6B inhibited metastasis of breast cancer cells probably by regulating the Wnt/β‐catenin signaling pathway.

## Conclusions

Our study concluded that KDM6B was lowly expressed in breast cancer tissues and correlated with the poor prognosis of patients with breast cancer. Furthermore, overexpression of KDM6B inhibited the proliferation, migration and metastasis of breast cancer, which might be mediated by regulating the Wnt/β‐catenin signaling pathway.

## Conflict of interest

The authors declare no conflict of interest.

## Data accessibility

The data in this study are available from the corresponding author upon reasonable request.

## Author contributions

QZ designed the study and analyzed data; JX and RG performed the study and wrote the manuscript; BW, YL, YM and JG assisted in carrying out the experiments. All authors have approved the final manuscript and have full access to all the data. All authors take responsibility for the integrity and security of the data.
